# A review on bigonial width reduction by botulinum toxin injections in masseter

**DOI:** 10.6026/97320630019272

**Published:** 2023-03-31

**Authors:** Anjali Sharad Ghatge, Sharad Balasaheb Ghatge, Vini Mehta

**Affiliations:** 1 Consultant Aesthetic Dermatogy, The Apollo Clinic, Mumbai 400001, India; 2 Division of Interventional Radiology, Department Of Radiology and Imaging, Grant Government Medical College and Sir JJ Group of Hospitals, Mumbai, Maharashtra, India; 3 Department of Interventional Neuroradiology, Bombay Hospital, Mumbai, Maharashtra, India; 4 Department of Public Health Dentistry, Dr. D.Y. Patil Dental College and Hospital, Dr. D.Y. Patil Vidyapeeth, Pimpri, Pune 411018, India

**Keywords:** Masseter muscle injections, botulinum toxin injection, bigonial width reduction

## Abstract

It is of interest to study the efficacy of botulinum toxin injection in masseter reducing bigonial width of the face. Online databases such as PubMed, Cochrane Library, Google Scholar, Science Direct database and ResearchGate were searched from initiation until October 2022 using keywords such as "masseter muscle injections", "botulinum toxin," "bigonial width reduction," "masseter muscle hypertrophy," and "lower face contouring" All available retrospective as well as prospective studies, were included with specific weightage to the efficiency of Botulinum Toxin (BT) injection in masseter muscle and the technique, unfavorable events, and the extent of its effects. A total of 20 publications were acknowledged. All prospective studies were included. Relevant data on patient selection, injection methods, efficiency, dose, rate of recurrence, and significant side effects of BT injections in masseters were collected. All excluding one were prospective studies. Bilateral injections were used in all the studies. Follow up period was 3-48 months. This systematic review focuses on the effectiveness of Botulinum toxin injection in lowering the bigonial width by decreasing the volume of the masseter. Repeat treatment at regular intervals is advisable to maintain desired results. The procedure was carried out in an office environment and was completed within 15 mins. There are no permanent adverse events related to the procedure. Further studies involving randomized control trials with larger sample sizes are warranted to optimize the dose and frequency of BT injection.

## Background:

The popularity of aesthetic procedures is growing rapidly. Shapely jawline, high cheekbones and carved-out hallows are the contemporary beauty trends. We want to be like the attractive faces of fashion models who stare at us from billboard neon boards and flamboyant movie screens. Reducing the bigonial width of the face improves the golden's PHI ratio and makes a face aesthetically appealing. Previous osteotomy of the jawbone, surgical resection of the masseter and liposuction of the hypodermic fat overlying the masseter were some techniques used to reduce bigonial width. However, these techniques were unpopular due to the severe adverse effects of surgical procedures and the ineffectiveness of percutaneous procedures [1,2]. There is a tectonic shift towards minimally invasive treatment options in a fast-changing world. Sculpting a V-shaped lower face under the zygomatic arch by percutaneous injections instead of surgery will be the crowning accomplishment. Botulinum toxin (BT) is widely utilized to reduce the mass and volume of the masseteric muscle, consequently decreasing the lower face's bigonial breadth. [3,4]. How far BT injections are effective in bigonial width reduction and sustainable patient satisfaction needs to be answered by systematic studies.Broad bigonial width is undesirable irrespective of gender preferences. The lower facial breadth is influenced by the size of the bony mandible, the mass of the masseter muscle, and the proportion of hypodermic fat tissue. The size of the mandible is determined by genetic structure. It varies with different races and ethnicity. It can be modified by invasive surgical osteotomy. The amount of subcutaneous fat depends on the individual body mass index and can be altered by weight management, diet, and surgical resection. The mass and width of the masseter muscle are also affected by personal habits like chewing, bruxism, food choices, biting force and/or disorders of the temporomandibular joint [5]. In both East Asian and Western cultures, BT injection into the masseter muscle is a efficacious and minimally invasive method for reshaping the lower jaw. [6]. Botulinum toxin has anatomic and physiologic effects on treated muscles. Aesthetic effects are due to anatomical modifications. Physiological effects though undesirable but unavoidable and are responsible for most of the complications [7, 8]. This review tries to answer the safety efficacy and durable benefits of BT injections in Asian and Western populations.

## Methods:

The systematic review was carried out in accordance with the Preferred Reporting Items for Systematic Reviews and Meta-Analyses (PRISMA) statement rules. [9] The key objective was to determine the effectiveness of BT injections in lowering masseter muscle mass. Secondary purpose was to see adverse effects and methods to avoid them by appropriate techniques and maintain the desired outcomes for a longer duration to the best patient satisfaction.

## Evidence acquisition:

The systematic review was planned with the electronic search of online databases such as PubMed, Cochrane Library, Google Scholar, Science Direct database and ResearchGate from beginning till September 2022 using keywords and Boolean operators such as "AND" and "OR". The keywords such as "masseter muscle injections", "botulinum toxin," "bigonial width reduction," "masseter muscle hypertrophy," and "lower face contouring" with a mixture of medical subject heading (MeSH) and free text terms were utilized. Articles published in the English language were considered. All accessible retrospective and prospective studies, case series, and case reports were evaluated, with a focus on the efficacy and safety of Botulinum toxin (BT) injections, as well as procedural parameters such as technique, BT dose, injection durability, and undesirable events. Additional unpublished data presented at scientific conferences or part of the authors' current experience was also included to accommodate the most recent information on contemporary techniques despite the bias introduced by such methodology.

## Screening and selection:

Two reviewers went over each piece individually, starting with the title and abstract. If the keywords appeared in the title or abstract, the articles were selected for full text reading. Papers with no abstracts but titles implying connection to the review's objectives were also selected to screen the whole text for eligibility. Following selection, two reviewers reviewed the full-text papers attentively. Data was extracted from articles that satisfied all of the entry requirements. Two reviewers searched the reference lists of all chosen papers for additional relevant literature. Disagreements among reviewers were resolved by mutual dialogue. If there was still a disagreement, the decision of a neutral third reviewer was considered final.

## Data extraction:

Data on bigonial width reduction by botulinum toxin injections was gathered from the numerous research that matched the inclusion criteria. For papers with insufficient data, the associated authors were contacted to get further information. The information was extracted individually by the two authors using specially designed data extraction forms built in Microsoft Excel software. All differences were resolved by dialogue among the writers. The following information was extracted from a standard form (when available) for each selected study: author and year of publication, study methodology, number of patients, male (sample size), female (sample size), demographic group, overall effectiveness, type of BT, max dose (units) per side per session, follow-up in months, evaluation, and side effects.

## Results:

## Search and selection studies:

**Figure 1:**
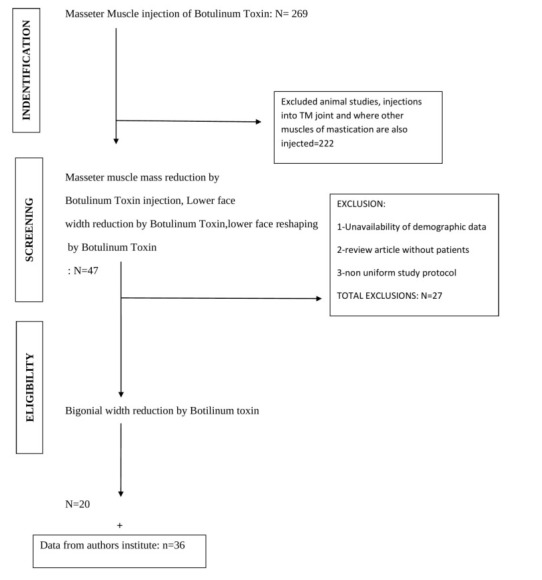
Flowchart illustrating the article selection procedure

This review evaluated 269 papers, of which 222 research involving injections into numerous masticatory muscles, TM joint injections, and animal experiments were discarded. The second level elimination of 27 articles was done based on the unavailability of demographic data and review articles. 20 studies were considered for this review. Unpublished study conducted in the author's institution was also included (Figure 1).

Data from included studies were summarized in tabular form (Table 1). All except one were prospective studies. Bilateral injections were used in all the studies. Studies with unilateral injections were excluded. Follow up period was 3-48 months. Due to the heterogeneity of the collected data, numerous perplexing factors, an astounding number of unpowered studies, and a deficit of homogenous control groups, a convincing statistical meta-analysis on the safety and efficacy of BT injections into masseter muscle for bigonial width reduction could not be conducted. Consequently, this study decided to cover salient topics in the following section with a focus on modern percutaneous injection techniques that may increase the odds of good clinical results.

## Discussion

Understanding the topographic connection of the facial nerve branches, facial artery and vein, parotid gland, and duct can aid in the prevention of disorders [10]. One of the muscles of mastication, the masseter is divided into two sections: deep and superficial. Because the needles used are so minute, the masseteric nerve and artery pass between these heads unaffected by the therapy. The zygomatic arch is connected to the top muscle boundary. The muscle's bottom margin is the lower border of the mandible. The posterior and anterior margins of the teeth are visible when the patient clenches his or her teeth. A line needs to be drawn from the angle of the mouth to the tragus, which acts as the safe limit decidedly. To prevent substantial dissemination to the pterygoid muscle via the coronoid notch and over-hollowing the superior portion of the muscle, Botox should be administered below this line. Diffusion of BT in lateral and medial pterygoid muscles can lead to restricted chewing ability [4]. This can also be avoided with smaller doses at each session. The parotid glands are a pair of salivary glands situated at the posterior edge of the mandible. The facial artery and vein run in front of the anterior boundary of the masseter [11].

Judicious selection of the patients is key to successful treatment outcomes. Patients should be informed about procedural details, expected outcomes, adverse effects, and frequency of repeat treatment. Typically, patients ages ranging from 15 to 40 years will solicit this treatment. However, the author's institute has seen patients up to the age of 50 years seeking this treatment. Older patients with sagging jowls are not suitable candidates, as BT injection into the masseters can deteriorate skin redundancy by reducing the muscle mass. Candidates with squared mandible, high cheekbones and excess subcutaneous fat should be explained about the alternative surgical or interventional treatment options [12]. Patients with bruxism and certain chewing habits may need additional treatment sessions to achieve an optimum result [13]. Broad bigonial width due to hypertrophied masseters is an ideal candidate for this treatment. [13, 14] Benign Masseter hypertrophy should be accurately diagnosed. The contraindications for BT injections are the conditions like tumours of the masseter muscle, parotid gland, inflammatory disease of the cheek and salivary gland. Subjective evaluation of the lower face is accomplished via inspection and palpation of the masseter muscle at rest and during clenching. Pre-treatment clinical photographs taken can be compared with post-treatment photographs. Objective assessment of pre and post-treatment bigonial width measurement is done by caliper. The thickness of the masseter can be measured by Ultrasonography (US), Computerized Tomography (CT) and magnetic resonance imaging (MRI). EMG carries out the electrophysiological assessment.

The following approaches for BT injections are suggested in this review. Lindernet al employed a one-two-point injection technique, with one injection at the angle of the jaw and the second in the area of the zygomatic arch [15]. A 2-point injection approach, 1 cm apart in the bottom one-third of the muscle, may also be employed [16, 17]. The 2-three-point injection method incorporates one site inferior to the TM line at the thickest point of a muscle and two spots 1cm apart from the anterior and posterior borders of the masseter. This reduces the possibility of damaging the risorius muscle or inducing a parotid gland herniation [18, 19]. Kim HJ et al. adopt a 3-four point approach, with two injection locations 1 cm apart indicated on a line from the tragus to the corner of the mouth. Another two injection locations are provided one centimetre above and below this reference line [20]. 4-Five or six-point Technique: 5-6 points with the borders of muscle in a grid-like fashion, each 1cm apart [21]. 6-Ultrasonography guided injection technique A new technique uses ultrasound-guided injections at 2-4 points, 1.5 cm apart within the borders of the muscle and tragusmouth line [22]. The researcher favours a three-point injection approach, with the first injection at the location of highest muscle bulging. The second and third spots are one centimetre below the first and one centimetre apart from the muscle's anterior and posterior edges, creating a triangle. A reconstituted lyophilized vial of BT with 2.5 ml of sterile, preservative-free 0.9% Sodium Chloride Injection was used. It was poured into a 1ml syringe. A 30G needle attached to a 1 cc syringe was used to inject the fluid. Each spot received 10 units of Botox, for a total of 30 units per side per masseter. [4] Injecting 1 cm away from the anterior border of the masseter minimizes the chances of paralyzing the risorius muscle [18]. It is better to inject at multiple sites than one to ensure uniform reduction in the size of the masseter. The procedure was carried out in an office environment and was completed within 15 mins.

Though BT injection was reported in 1994, it evolved. Initial studies showed the safety and efficacy of BT injections in masseter hypertrophy but were restricted due to smaller sample sizes, variable doses, and shorter investigation periods [21, 23,24]. Later, studies with larger sample sizes and lengthier follow-up periods optimized treatment protocols. Kim and colleagues used BT to treat 1021 Korean patients for masseter volume reduction in 1 to 2 treatment sessions at 5-month intervals. The thickness of the muscle was decreased by 31% on average after a 3-month follow-up (n = 383), as measured by ultrasonography. The volume loss continued even after muscle function was restored [20]. Kim and colleagues (n = 11) and Yu and colleagues (n = 10) conducted CT scans to quantify muscle size and reported a comparable decrease of 22% and 30% at 3 months following 1 session of BT therapy [5, 8]. The optimum dose of BT is yet to be defined, but most studies show 20-40 units of botulinum, considering equivalent doses for various types.[25] It is amply proved doses less than 20 units of BT are inadequate [26]. Factors like the type of BT, racial variation, gender, muscle thickness, ethnicity and cultural differences determine doses. Dysport preparations containing Abobotulinumtoxin A require relatively larger doses [8]. Determining the Optimal frequency of injections is still under investigation and will evolve with time. Kim and colleagues medicated 121 Korean patients over 52 months with a series of 1 to 8 injections spaced at 1 to 19 month intervals. The scientists concluded that the dosage of BT dropped with the frequency of visits, and the mean thickness of the masseter muscle reduced with increasing treatments. There were no adverse effects recorded in this trial. To maintain desirable outcomes, treatments should be repeated 2 to 4 times each year [13,14].

There are other varieties of BT, but only Types A and B are permitted for therapeutic usage. There are many variants of Botulinum Toxin A, including Abobotulinumtoxin A, Incobotulinumtoxin A, Onabotulinumtoxin A, and Prabotulinumtoxin A. Several subtypes of A toxins, including botulinum toxin, abobotulinum toxin, and Nabota, were individually compared to onabotulinum toxin in East and Southeast Asians. Given the same number of doses, all showed comparable effectiveness [27, 28,29]. Rimabotulinum toxin B was compared to onabotulinum toxin A in another research in Korean women. After one injection, both exhibited a similar decrease in masseter volume, although rimabotulinum toxin B had a shorter action period than onabotulinum toxin A [30]. As a result, Botulinum toxin A is preferable over Botulinum toxin B. There is no evidence to prove the efficacy of one toxin over the other [31]. Abobotulinumtoxin A (ABO) to Incobotulinumtoxin A (INCO) to Onabotulinumtoxin A (ONA) conversion factor is roughly 3:1:1:1 or slightly lower [31]. A BT injection causes muscular paralysis. Paralyzed muscles atrophy over time owing to being overused or underutilised. Because BT-induced muscle paralysis is reversible, it is critical to provide BT injections at periodic intervals to sustain masseter mass decrease [10, 32,33].

The adverse effects of BT injections are due to their action on masseter muscle resulting in decreased mastication, altered facial expressions, and uneven muscle mass bulging. Mastication is reduced after 2-4 weeks of administration, peaks at 3 weeks, and lasts 8-12 weeks [1,12,8]. The reported incidence of this is approximately 30%. This adverse effect is expected and may not be completely avoidable. Painful bruising of the masseter is the second most common side effect amounting to 2.5%. Other adverse effects are below 1% [34]. Inadvertent injection into risorus causing paralysis leading to asymmetric facial expression is an unintended impediment. It can be completely avoided through a judicious method of selecting points of injections within the safest margins of the masseter [12]. Inappropriate location and deeper injections of BT may result in uneven bulging of muscles [35]. Osteopenic effects of botulinum toxin injections on the masticatory muscles are mostly seen in animal studies. One pilot study in humans raises concerns that need to be validated in larger human studies. Generally, BT injections are very safe, and in the authors opinion, there are no permanent side effects, and all other potential side effects are reversible.

## Conclusion:

This systematic assessment highlights the efficacy of Botulinum toxin injection in reducing the bigonial width by reducing the volume of the masseter. Repeat treatment at regular intervals is advisable to maintain desired results. The treatment is performed in an office environment and takes about 15 minutes. There are no permanent adverse events related to the procedure. Further studies involving randomized control trials with larger sample sizes are warranted to optimize the dose and frequency of BT injection.

## Figures and Tables

**Table 1:** Characteristics of the included studies

**StudyID**	**Studydesign**	**Samplesize**	**Male**	**Female**	**SuccessRate**	**TypeofBT**	**MaxDose(Units)persidepersession**	**Followupinmonths**	**Agegroup**	**Assessment**	**TemporaryComplications**
SmythAG*etal*(1994)[24]	prospective	7	3	4	100	ABO	300	12	17-23	EMG	LP,B
TOEW*etal*(2001)[34]	prospective	5	4	1	100	ONA	150	12	16-32	US	none
kim*etal*(2003)[20]	prospective	11	2	9	90	ONA	30	12	25-45	CT	DM,SC,AS,GD
Park*etal*(2003)[1]	prospective	45	2	43	89	ONA	30	12	24-48	US,CT	LP,DM,FA
ChoeSW*etal*(2005)[27]	prospective	22	0	10	100	ONA	30	9	21-35	US	LP,HA
Kim*etal*(2005)[5]	prospective	383	28	355	100	ABO	140	3	15-40	US	DM,FA
Arikan*etal*(2006)[2]	prospective	5	2	3	100	ABO	####	8	19-35	Clinicalphotograph,CT	none
Kim*etal*(2007)[26]	prospective	32	14	18	100	ONA	35	3	22-36	CT,EMG	HA,DM,LP,X
Chung-ChihYu*etal*(2007)[8]	prospective	10	0	10	90	ABO	120	12	25-46	CT	DM,SC
LiewandDart*etal*(2008)studygroup[7]	prospective	34	0	34	100	ONA	30	18	21-52	ClinicalPhotograph,patientfeedback	B,FA,S
LiewandDart*etal*(2008)controlgroup[7]	prospective	48	0	48	100	ONA	45	24	19-35	ClinicalPhotograph,patientfeedback	DM
TartaroG*etal*(2008)[9]	prospective	5	2	3	100	ONA	50	12	46-56	EMG	none
Kim*etal*(2010)[12]	retrospective	121	0	121	100	ABO	140	12	17-51	US	none
Shim*etal*(2010)[17]	prospective	15	4	11	100	ONA	25	24	22-35	3Dlaserscan	none
KleinFH*etal*(2014)[3]	prospective	10	0	10	100	ONA	90	6	25-40	ClinicalPhotograph	DM,FA
Xie*etal*(2014)[10]	prospective	220	15	205	95.91	ONA	40	4	20-40	US,clinicalphotograph	B,DM,HA,SC,AS
NikolisA*etal*(2018)[33]	prospective	30	0	30	100	ONA	40	18	19-57	Clinicalphotograph,patient	LP,B
Chun-shinchang*etal*(2019)[19]	prospective	6	0	6	100	ONA	24	12	21-39	3DCT	none
Shome*etal*(2019)[13]	prospective	50	29	21	100	ONA	30	48	21-60	US,clinicalphotograph	LP,HA
Shome*etal*(2020)[14]	prospective	30	15	15	100	ONA	30	12	24-55	USclinicalphotograph	LP
Authorsdata	retrospective	36	12	24	100	ONA	30	6	18-52	Clinicalphotograph,Bigonialwidthmeasurementbycalipre	B,LA,DM,
CT-Computed tomography, EMG-Electromyography, US-Ultrasound, DM- Decreased Mastication,LP-Local Pain, FA-Facial Asymetry, B-Bruise, HA-Headache, S-Sagging Skin, SC-Sunken Cheeks, AS-Abnormal Smile,GD-Gustatory Difficulty, X-Xerostomia, ABO- Abobotulinum Toxin, ONA-Onabotulinum Toxin, INCO-Incobotulinum Toxin
